# The Vein Patterning 1 (VEP1) Gene Family Laterally Spread through an Ecological Network

**DOI:** 10.1371/journal.pone.0022279

**Published:** 2011-07-26

**Authors:** Rosa Tarrío, Francisco J. Ayala, Francisco Rodríguez-Trelles

**Affiliations:** 1 Grup de Biologia Evolutiva, Departament de Genètica i de Microbiologia, Universitat Autònoma de Barcelona, Barcelona, Spain; 2 Universidad de Santiago de Compostela, CIBERER, Genome Medicine Group, Santiago de Compostela, Spain; 3 Department of Ecology and Evolutionary Biology, University of California Irvine, Irvine, California, United States of America; East Carolina University, United States of America

## Abstract

Lateral gene transfer (LGT) is a major evolutionary mechanism in prokaryotes. Knowledge about LGT— particularly, multicellular— eukaryotes has only recently started to accumulate. A widespread assumption sees the gene as the unit of LGT, largely because little is yet known about how LGT chances are affected by structural/functional features at the subgenic level. Here we trace the evolutionary trajectory of VEin Patterning 1, a novel gene family known to be essential for plant development and defense. At the subgenic level VEP1 encodes a dinucleotide-binding Rossmann-fold domain, in common with members of the short-chain dehydrogenase/reductase (SDR) protein family. We found: i) VEP1 likely originated in an aerobic, mesophilic and chemoorganotrophic α-proteobacterium, and was laterally propagated through nets of ecological interactions, including multiple LGTs between phylogenetically distant green plant/fungi-associated bacteria, and five independent LGTs to eukaryotes. Of these latest five transfers, three are ancient LGTs, implicating an ancestral fungus, the last common ancestor of land plants and an ancestral trebouxiophyte green alga, and two are recent LGTs to modern embryophytes. ii) VEP1's rampant LGT behavior was enabled by the robustness and broad utility of the dinucleotide-binding Rossmann-fold, which provided a platform for the evolution of two unprecedented departures from the canonical SDR catalytic triad. iii) The fate of VEP1 in eukaryotes has been different in different lineages, being ubiquitous and highly conserved in land plants, whereas fungi underwent multiple losses. And iv) VEP1-harboring bacteria include non-phytopathogenic and phytopathogenic symbionts which are non-randomly distributed with respect to the type of harbored VEP1 gene. Our findings suggest that VEP1 may have been instrumental for the evolutionary transition of green plants to land, and point to a LGT-mediated ‘Trojan Horse’ mechanism for the evolution of bacterial pathogenesis against plants. VEP1 may serve as tool for revealing microbial interactions in plant/fungi-associated environments.

## Introduction

The existence of specialized mechanisms of genetic transfer between bacteria was known decades before the advent of genomics [1]. However, the evolutionary significance of genetic flux and mobile genetic elements –the so-called *mobilome* [2]–[4]— started to be fully appreciated only after the accumulation of i) patterns of gene presence or absence that could not be reconciled with a pattern of strict vertical descent, and ii) topological discordances between gene trees, or between gene trees and trusted reference trees [5]–[7]. It is now clear that the most diverse and ubiquitous life forms on Earth, namely viruses and microbes, exhibit levels of lateral gene transfer (LGT, also known as horizontal gene transfer, or the non-genealogical transmission of genetic material from one organism to another [8]) that question the adequacy of the “Tree of Life” as an overarching metaphor of evolutionary history [5], [9]–[10].

LGT detection is usually best tackled by adopting a phylogenetic approach [11]–[13], which for recent events can be buttressed on non-phylogenetic, so-called surrogate approaches, such as biased nucleotide base composition [6], [13]. Analyses with these methods, especially since the dawn of genomic technologies, have shown that LGT i) can involve virtually any sequence, from few-nucleotide-long tracts to entire chromosomes [6], [14]–[15]; ii) can take place between any taxa, regardless of their phylogenetic distance, and in every possible direction [11], [13], [16], yet it does not occur indiscriminately; iii) appears to be far more frequent within and between Bacteria and Archaea, and from these taxa to unicellular eukaryotes than to or between multicellular eukaryotes –perhaps because in multicellular eukaryotes the germ line acts as a physical barrier against foreign DNA, or the regulatory networks are more complex, which would make integration more difficult [13]; iv) is more frequent between organisms sharing the same habitat than between ecologically unrelated organisms [17]–[18]; v) can affect any gene [10], [19], so that it is estimated that the typical prokaryotic gene family undergoes a minimum average of 1–2 LGT events over its full evolutionary lifespan [20], [21]; and vi) successful LGTs are biased toward roles that are directly related to specific environmental conditions, such as defense and pathogenicity, aerobiosis or limiting-nutrient uptake [18], [22]–[25].

Despite these and many other advances, knowledge about rates and patterns of LGT involving eukaryotes remain largely tentative, owing to the limited availability of complete genomes [11]–[13], [26]–[28]. Most functional transferomics analyses set off from the gene as the unit of LGT. Recent studies relaxing this assumption have not found evidence that LGT-associated recent recombination events respect the integrity of sequences encoding protein domains [14], [15]. But the possibility has been noted that functional domains may have modular structures, consisting of functional sub-domains irregularly distributed along the primary sequence [14]. For example, the classical Rossmann dinucleotide-binding domain, one of the oldest and most pervasive folds in nature has been recently shown to be organized in this way [29]. Modular functional encoding is expected to confer mutational robustness, hence enhanced potential for functional innovation [30], [31], but there is little empirical knowledge about how this property relates to the likelihood of successful LGT. The issue is particularly relevant for long-distance LGT, considering the potential for dramatic genetic rearrangement associated with semi-homologous and/or illegitimate recombinational mechanisms [3]. The proven ability of LGT for transferring phenotypes makes it an ideal candidate for being instrumental in rapid evolutionary transitions, such as the colonization of land by plants and fungi or the shift to a pathogenic lifestyle [18], [32]–[37]. But the search for key adaptive LGT genes has only started. Herein, we characterize the origin and evolutionary history of VEin Patterning 1 (VEP1), a novel protein gene family at the crossroads of these questions.

Three separate lines of inquiry have coined three different names for the same orthologous gene (locus at4g24220 in *Arabidopsis*). The first line involved the pathway for the biosynthesis of cardenolides in foxglove (*Digitalis* genus) [38], [39], [40], [41]. Also known as cardiac glycosides or cardiotonic steroids, cardenolides are plant defense secondary metabolites of great pharmacological interest, owing to their long time use to treat cardiac insufficiency in humans. Work along this line identified a gene sequence encoding progesterone 5β-reductase activity *in vitro*, thereby P5βR was proposed to be the catalyst of the first committed step of the cardenolide pathway *in vivo*. Recently, it was found that the P5βR gene i) is not exclusive to the foxglove, but is also present in cardenolide nonproducing plants [41], [42]; ii) is evolutionarily unrelated to its putative functional homolog in animals [41]; and iii) the enzyme shows greater affinity for some small non steroid substrates than for progesterone in vitro [43]. The second line of research concerned the genetics of plant responses to stress. A screening of an *Arabidopsis* cDNA library constructed from the plant tissues upon wounding treatment resulted in the isolation of the AWI 31 (*Arabidopsis* Wound Inducible 31) gene [44]. The third line focused on the genetic dissection of plant vascular development. A random antisense mutagenesis experiment in *Arabidopsis* discovered that antisense suppression of a gene, then called VEP1, causes drastic reduction in the complexity of the leaf venation pattern [45]. The present study adopts the VEP1 name, because in our view it establishes a most definite functional link for the gene. Altogether, the aforementioned evidence (plus novel features highlighted later) hints that VEP1 pertains to a category of essential genes, which are required for plant growth and development, and have also important functions in defense [46]. Structurally, VEP1 encodes a single domain protein consisting of a Rossmann dinucleotide-binding fold, which is evolutionarily related to the short-chain dehydrogenases/reductases (SDRs), but with an unprecedented active site [41], [47], [48].

## Materials and Methods

### Reference tree topology, VEP1 gene presence/absence, and sequence data

The reference (species) tree topology is a consensus of trees from various sources, including NCBI taxonomy [49], ‘Tree of Life’ [50], ‘The All-Species Living Tree’ project [51], and TIMETREE [52]. VEP1-containing lineages were identified by performing homology searches using the BLASTp and tBLASTn tools [53] against the NR, EST, WGS, GSS, and HTGS databases at the National Center for Biotechnology Information (NCBI; http://www.ncbi.nlm.nih.gov/). In order to improve taxonomic coverage for gene presence, additional specialized genome databases were considered, including the DOE Joint Genome Institute databases (Genome Portal, Phytozome, and Integrated Microbial Genomes) (http://www.jgi.doe.gov/), the Fungal Genome Initiative database (http://www.broadinstitute.org/science/projects/fungal-genome-initiative/fungal-genome-initiative), the TIGR Plant Transcript Assemblies database (http://plantta.jcvi.org/), and the Dragonblast web tool (http://dbdata.rutgers.edu/dragon/). Identification of VEP1-lacking lineages requires knowledge of complete genomes. VEP1 absence in a lineage was inferred when homology searches against the corresponding genome resulted in no significant hits. Unless stated otherwise, close homologs exhibiting pairwise amino acid sequence identity ≥25% and query coverage ≥90% in the BLAST output were considered for gene tree reconstruction. An initial data set of 81 amino acid sequences was selected, including five representatives from each one of fungi and Embryophyta, the only ones found in Chlorophyta, and all detected bacterial sequences to the species name level.

### Multiple sequence alignment (MSA) and phylogenetic inference

Protein structures evolve more slowly than their sequences [54]. Structure-based MSA methods are expected to be more accurate than sequence-only-based MSA methods. There is currently a three-dimensional (3D) crystal model of a homolog of the target protein from the plant *Digitalis lanata* in the Protein Data Bank (PDB codes *2v6f*-*g*). Structure-based MSA of VEP1 sequences was conducted using regular EXPRESSO (3D-Coffee) (http://www.tcoffee.org/) [55], which automatically fetches *2v6f* to guide the structural MSA. Taking into account the current EXPRESSO operational limit of up to 50 sequences per batch, the MSA workflow was divided into three steps: first, reduction of the initial 81 sequences data set to a core set of 50 least redundant sequences, using the ‘Decrease Redundancy’ tool from the Expasy Proteomic Server (http://expasy.org/tools/redundancy/), setting maximum identity to 70%. Second, structural MSA of the core set using EXPRESSO. Third, alignment of the 31 sequences excluded from the core set in step one to the EXPRESSO MSA one at a time, using the ‘sequence-to-profile’ option of CLUSTALW with manual refinement. Reliability of the positional homology inference was color-coded using the T-Coffee CORE (Consistency of Overall Residue Evaluation)–index [56]. The majority of residues in the *2v6f* structure guided-MSA of VEP1 were in the average-to-good range, and the MSA received a CORE index score of 91, where a score ≥50 indicates a 90% probability of being correctly aligned [57]. Prior to phylogenetic inference, the MSA was masked to remove ambiguous alignment positions using the Gblocks server (http://molevol.cmima.csic.es/castresana/Gblocks_server.html) [58] with each of the options for less stringent selection chosen [59]. The resulting MSA retained 239 columns.

A model-based maximum-likelihood (ML) framework of statistical inference was adopted for tree reconstruction. First, the amino acid replacement process of the VEP1 gene was modeled using an initial tree topology that is approximately correct; then, the best-fit model was used to search for the ML gene tree. Amino acid replacement modeling was conducted automatically using the ProtTest server (http://darwin.uvigo.es/software/prottest_server.html) [60] with default options. The best description of the amino acid replacement process of the VEP1 gene was provided by the LG+F+dG+I model, which incorporates the empirical replacement matrix of [61] (LG component), amino acid frequencies set as free parameters (F), four categories of gamma distributed rates across sites (G), and a proportion of invariant sites (I). Heuristic search of ML trees was conducted using PhyML v3.0 [62], starting from a BioNJ distance-based tree, with the best of NNI (Nearest Neighbor Interchange) and SPR (Subtree Prunning and Regrafting) tree topology search methods. Branch support was estimated using 1000 non-parametric bootstrap pseudoreplicates, and the approximate likelihood ratio test (aLRT [63]), with statistical significance calculated by the Shimoidara–Hasegawa-like (SH-like) non-parametric method [64].

### Lateral gene transfer analysis

Analyzing the 81 taxa of this study for LGT simultaneously would yield too many LGT events. For simplicity, we considered intradomain (i.e., among bacteria) LGT separately from interdomain (i.e., bacteria-to-eukaryota) LGT, and conducted the intradomain LGT analysis separately for each bacterial cluster (Clusters I, IIa, and IIb). The direction of intradomain LGT was inferred using the LGT-detection tool [65] at the T-REX server (http://www.trex.uqam.ca/). This tool works by progressive reconciliation of the given rooted species and gene topologies using SPR moves (*i.e.*, LGTs). Bipartition dissimilarity (BD) was adopted as optimization criterion for the searching of optimal SPR scenarios. Reliability of obtained LGTs was assessed by non-parametric bootstrap analysis [65], holding constant the species tree against 1000 gene trees, each generated from a pseudoreplicate of the original alignment by the same inferring method used to construct the original gene tree as described above. In a species tree with the form ((*a*,*b*),*c*), in which *a*, *b* and *c* may respectively represent plant, fungi and bacteria as in this study, opposite LGTs *a*→*c* and *c*→*a* lead to the same topological rearrangement, i.e. ((*a*,*c*),*b*). In situations like this, current LGT detection methods are not guaranteed to identify the correct LGT scenario [65]. Therefore, in the present work the direction of interdomain LGTs was inferred based on the relative distribution of the gene among bacteria and eukaryotes (*e.g.*, [66]), rather than on topological discordance between species and gene trees.

### VEP1's closest remote homolog identification and evolutionary structural analysis

Search for distantly related homologs was conducted using numerical and probabilistic profile-based methods, and structure-based methods. Position Specific Iterated-BLAST (PSI-BLAST) [67] five iteration-runs with default parameters were used to search the NR protein sequence database. PSI-BLAST false discovery rates were controlled using SIB-BLAST [68], which benchmarks PSI-BLAST last iteration's hits against those from the second iteration when the profile (Position Specific Score Matrix; PSSM) is expected to be least corrupted. PSI-BLAST–based COMPASS [69], and/or profile Hidden Markov Model (HMM)–based Profile Comparer (PRC) [70] were used to search against corresponding sequence profile libraries, including Pfam [71], SCOP [72] and SUPERFAMILY [73], and COGs and KOGs [74]. Query profiles for profile HMM-based searches were built with the HMMER vs3.0 [75]-based HMMbuild tool at the Mobyle Portal (http://mobyle.pasteur.fr/) using herein inferred MSA as input. Structural similarity searches of the Protein Data Bank (PDB) were performed using DaliLite v.3 [76] using *D. lanata*'s *2v6f*-*g* PDB structures as queries. Multiple structural alignment and superposition of distantly related structures, Root-Mean Square Deviation (RMSD)-based molecular sieving, and corresponding Lesk-Hubbard plots were performed using the MUSTANG-MR server [77]. Graphical representations of the patterns within MSAs were obtained with WebLogo [78]. VEP1 three-dimensional images were generated using DeepView [79].

## Results

### Distribution of the VEP1 gene across the reference tree

**Figure 1:**
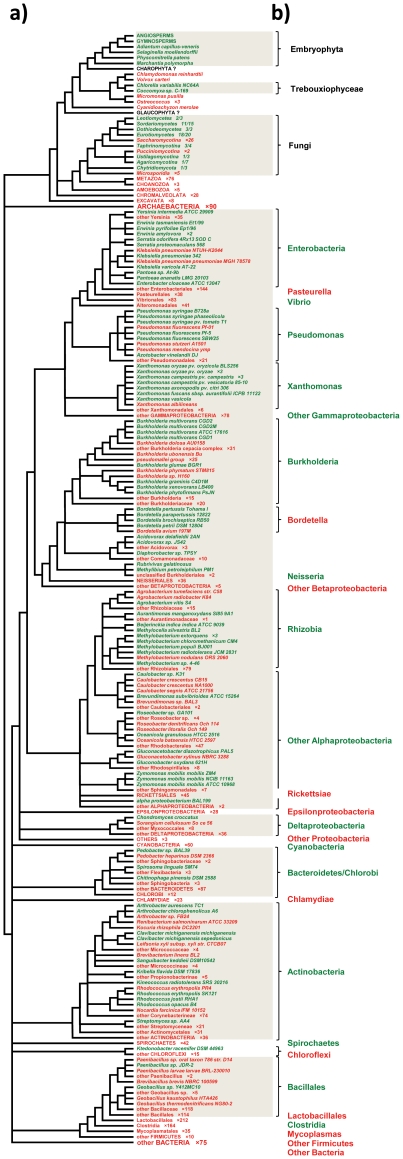
Presence (green)/absence (red) distribution of VEP1 across the reference tree. The reference tree topology is based on information from various sources, including NCBI taxonomy [49], ‘Tree of Life’ [50], ‘The All-Species Living Tree’ project [51], and TIMETREE [52] (see the Materials and Methods section).

Figure 1 shows the distribution of VEP1 across the reference tree, with the species colored green/red to denote presence/absence of the gene. VEP1 is a rare gene, which exhibits a broad, yet extremely spotty phylogenetic pattern of occurrence. The gene is present in Bacteria and eukaryotes, but absent in Archaea. Of the 26 bacterial phyla with at least one completed genome at NCBI's Microbial Genomes (http://www.ncbi.nlm.nih.gov/genomes/MICROBES/microbial_taxtree.html), VEP1 is present only in five, namely Actinobacteria, Bacteroidetes, Chloroflexi, Firmicutes and Proteobacteria. The gene is absent in Chlamydiae, Cyanobacteria, Fusobacteria, Spirochaetes, and Tenericutes, although these phyla exhibit relatively ample coverage of genome projects. The phylum with the greatest number of VEP1-containing genera is Proteobacteria. VEP1 is present only in two Firmicutes (*Geobacillus sp*. Y412MC10 and *Paenibacillus sp*. JDR-2), in spite of this being the second phylum with the greatest number of available genomes after Proteobacteria. Within Proteobacteria, VEP1 was found in Beta-, Gamma-, Alpha-, and Deltaproteobacteria, but the distribution of the gene within each of these four classes is extremely spotty. For example, of eight betaproteobacterial orders VEP1 is found only in *Burkholderiales*. Within this order the gene is present in four strains of *Burkholderia multivorans* (CGD1, CGD2, CGD2M, and ATCC 17616), and in *B. glumae* BGR1, *B. graminis* C4D1M, *B. phytofirmans* PsJN and *B. xenovorans* LB400, but it could not be detected in any of 70 intermediate taxa, including *B. dolosa* AU0158 and 31 other representatives of the *B. cepacia* complex, *B. ubonensis* Bu, 35 representatives of the *pseudomallei* group, *B. phymatum* STM815, and *B. sp*. H160. Overall, the pattern of occurrence of VEP1 in Bacteria suggests an evolutionary history dominated by horizontal gene transfer and loss.

Within eukaryotes VEP1 was detected exclusively in green plants and fungi. The distribution of VEP1 within green plants is discontinuous. Exhaustive tBLASTn searches against all publicly available sequence databases, including NCBI's dbEST and TIGR Plant Transcript Assemblies databases detected the gene in dicots and monocots, gymnosperms, the fern *Adiantum capillus-veneris*, the club-moss *Selaginella moellendorffii*, the moss *Physcomitrella patens*, and the liverwort *Marchantia polymorpha*. The gene could not be detected in basal Streptophya, despite the availability of EST libraries for representatives of Coleochaetales (*Coleochaete orbicularis*), Zygnematales (*Spyrogyra pratensis*), and Mesostigmatales (*Mesostigma viride*), which strongly indicates that the phylogenetic distribution of VEP1 in Streptophyta is restricted to embryophytes. Analogously, in Chlorophyta the gene could only be detected in two *Trebouxiophyceae* algae, namely *Chlorella variabilis* NC64A and *Coccomyxa sp*. C-169. None of the available Chlorophyceae (*Chlamydomonas reinhardtii* and *Volvox carteri*) and the more distantly related Prasinophyceae (three species of *Ostreococcus* and *Micromonas pusilla*) genomes yielded positive results. In fungi the occurrence of VEP1 is far less predictable than in land plants. Overall, the gene could be detected in representatives of the major phyla except the basal phylum Microsporidia. Within Ascomycota, VEP1 was detected in the subphyla Pezizomycotina and Taphrinomycotina, but not in every genome, and the gene is absent in all 26 available genomes of the subphylum Saccharomycotina. In addition, VEP1 was detected only in two out of 12 Basidiomycota species, namely *Ustilago maydis* (Ustilaginomycotina) and *Cryptococcus neoformans* (Agaricomycotina), and only in *Spizellomyces punctatus* out of three Chitridiomycota species. The increased spotty distribution of VEP1 in Fungi indicates that this phylum exhibits a decreased propensity for VEP1 retention (or acquisition?) compared to land plants. The restricted phylogenetic distribution of VEP1 in eukaryotes suggests that the gene was acquired in this domain via LGT from Bacteria. Yet land plants and the trebouxiophytes are distantly related to each other, and further apart from the fungal kingdom, which suggests that bacteria-to-eukaryote transfer of VEP1 might have occurred several times in evolution. If this was the case, then each lineage would be expected to cluster with a separate group of bacteria in the VEP1 gene tree.

### The VEP1 gene tree

**Figure 2:**
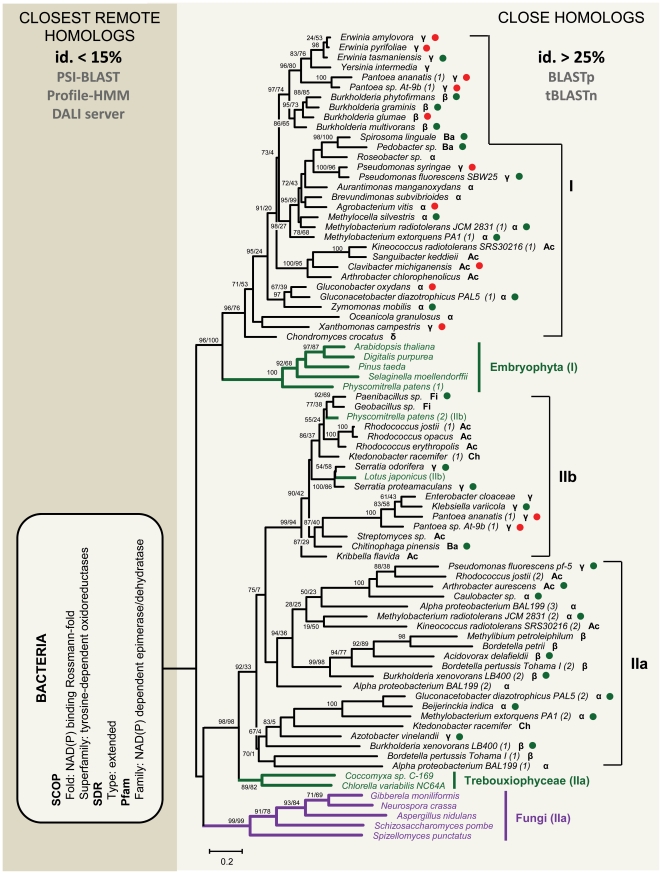
ML phylogenetic tree of VEP1. The tree was inferred from 239 amino acid characters using the empirical replacement matrix of [61], setting amino acid frequencies as free parameters, gamma-distributed rates among sites (4 categories; *α* = 1.532), and a proportion of invariant sites (I = 0.060), referred to as LG+F+dG+I model. Non-parametric bootstrap (1000 replicates)/aLRT support scores greater than 50% are shown above the respective nodes. Light (right) and dark (left) background areas indicate, respectively, the sequences used for building the tree (identified using tBLASTn; >25% pairwise sequence identity), and the extant closest remote homologs of VEP1 (identified using remote homology searching methods), which were not used for tree building, but are shown to indicate this study's hypothesis about the evolutionary origin of VEP1. Subtrees subtending inferred bacteria-to-eukaryote LGT events are colored green (viridiplantae) and fucsia (fungi). Green and red dots next to the taxa labels indicate plant-associated non-phytopathogenic and phytopathogenic bacteria, respectively. α, β, γ, and δ denote Alpha-, Beta-, Gamma, and Epsilon-proteobacteria, respectively; Ac, Actinobacteria; Ba, Bacteroidetes; Ch, Chloroflexi; Fi, Firmicutes.

Figure 2 shows the VEP1 ML gene tree. The tree incorporates all detected prokaryotic sequences to the species level, the only two Trebouxiophyceae BLASTp/tBLASTn positives, five representative least-redundant sequences from each of Embryophyta and Fungi, and two additional sequences including one from *Lotus corniculatus* and a second homolog from *Physcomitrella patens*, herein considered because their top BLAST hits were to Bacteria. It must be noted that the most distantly related homologs detected using the BLASTp and tBLASTn tools show a minimum ∼25% identity to the query. More remotely related homologs (≤15% identity; referred to as closest remote homologs in Figure 2) are reachable using profile-based and structure-based strategies. The sequences retrieved with these methods are primarily bacterial SDRs (see below), which is consistent with a bacterial origin of VEP1. Yet these sequences are too divergent to be used effectively as an outgroup. Besides this, the tree identified three bacterial clusters, which are denoted I, IIa, and IIb, with embryophytes resembling Cluster I, and fungi and the trebouxiophytes Cluster IIa (see below). The decision was conservatively made to place the root between bacterial clusters I and IIa based on reasoning that rooting the tree within bacterial cluster IIa, which is the most sequence-diverse and therefore could be presumed to be ancestral, would place the fungi between embryophytes and trebouxiophytes. But chytrids-Dikarya is the oldest eukaryotic node in the tree, conventionally assumed to be about twice as old as the diversification of land plants [52]. Note, however, that more intricate LGT scenarios that would follow from this alternative to the chosen root in Figure 2, involving eukaryote-to-eukaryote and eukaryote-to-bacteria transfers in addition to bacteria-to-eukaryote transfers, would not contradict the hypothesis set forth in this study.

Two main issues are apparent in Figure 2. First, as predicted if the distribution of VEP1 in the tree of life (Figure 1a) involved multiple interdomain LGTs, Embryophyta, the two trebouxiophyte algae, and the fungi do not form a monophyletic group but cluster separately, each offshooting from a different bacterial lineage with strong statistical support (aLRT and bootstrap values >95%). In total, the tree calls for five bacteria-to-eukaryote LGT events, namely: i) from an ancestor of bacteria IIa to the ancestor of chytrids-Dicarya; ii) from an ancestral bacteria I to the ancestor of land plants; iii) from an ancestral bacteria IIa to the ancestor of the trebouxiophytes; iv) from the common ancestor of *Paenibacillus sp*. JDR-2 and *Geobacillus sp*. Y412MC10 to *Physcomitrella*; and v) from an ancestor of *Serratia odorifera* 4R×13 SODc to *Lotus*. The statistical support for the nodes corresponding to LGTs iv) and v) is relatively weak, but in both cases the putative recipients clearly branch off from within bacterial Cluster IIb.

**Figure 3:**
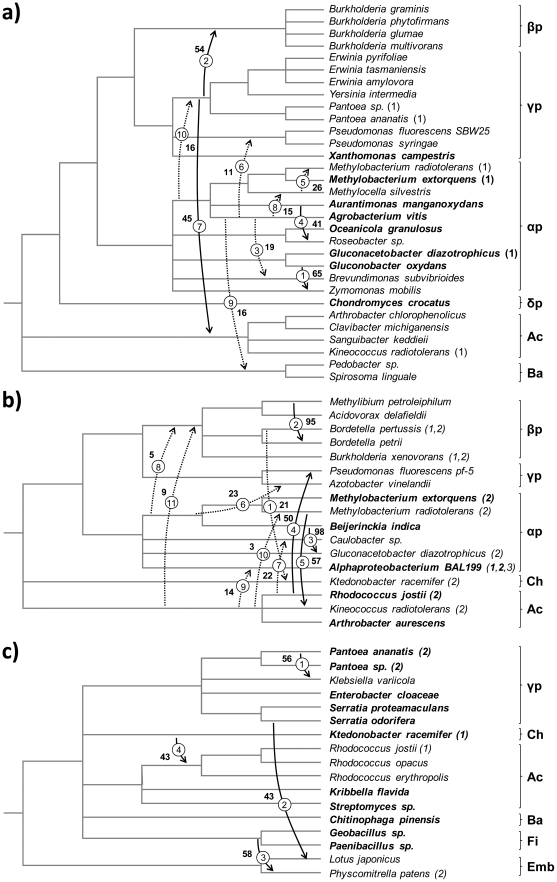
LGT scenarios for a) bacterial cluster I; b) bacterial cluster IIa; c) bacterial cluster IIb. The direction of LGT was inferred with the LGT-detection tool of the T-REX suite [65] adopting the bipartition dissimilarity optimization criterion. Non-parametric bootstrap (1000 replicates) scores are indicated near to the numbers (encircled) of the corresponding LGTs. Solid arrows denote inferred probable LGTs (bootstrap score >40%), and dashed arrows indicate possible LGTs (bootstrap score <40%). In bold are taxa inferred not to have obtained VEP1 through LGT. Numbers in parentheses next to taxon labels denote VEP1 copies in the corresponding cluster. For example, *Methylobacterium radiotolerans* has two VEP1 genes, the first (1) in cluster I (panel 3a), and the second (2) in cluster IIa (panel 3b), with the two copies acquired via LGT; Alphaproteobacterium BAL199 has three VEP1 genes (1, 2, 3), all in cluster IIb (panel 3b), of which gene number 3 was acquired via LGT. α, β, γ, and δ denote Alpha-, Beta-, Gamma-, and Epsilon-proteobacteria, respectively; Ac, Actinobacteria; Ba, Bacteroidetes; Ch, Chloroflexi; Fi, Firmicutes; Emb, Embryophytes.

Second, there are rampant inconsistencies within Bacteria between the phylogeny of VEP1 and the commonly accepted phylogeny of the species. Even when VEP1 is present in more than one copy in the same bacterium, LGT is the most likely origin of the extra copies. For example, the alphaproteobacterium *Methylobacterium radiotolerans* JCM 2831 and the actinobacterium *Kineococcus radiotolerans* SRS30216 each occurs in clusters I and IIa. The presence of two VEP1 copies in each of these bacteria is inconsistent with an ancestral duplication scenario, because the Proteobacteria-Actinobacteria split is much older than the diversification of the Viridiplantae, whereas in Figure 2 Clusters I and IIb are younger than the split Embryophyta-Chlorophyta. Figure 3a–c shows minimum cost LGT scenarios for each cluster, inferred using the LGT-detection method [65]. The total number of estimated LGT events is 25, of which 10 occurred in Cluster I (31 VEP1 genes), 11 in Cluster IIa (21), and 4 in Cluster IIb (17). The statistical support for the events is variable but low in general, which can be explained as a consequence of a combination of one or several factors (reviewed in [65]), including conservativeness of the bootstrap approach, a corresponding low bootstrap score in the original gene tree (e.g. score 65% of LGT number 1 in Figure 3a corresponds to score 97% in Figure 2), and a possibility of the opposite LGTs leading to the same topological rearrangement as that induced by the obtained transfer (e.g. LGT number 5 in Figure 3a). Be that as it may, it should be noted that Figure 2 includes all the VEP1-containing bacteria that were possible to detect at the time of this study, which means that VEP1 is a rare gene in Bacteria. This feature, together with the extremely spotty taxonomic distribution of the gene, and the rampant topological conflicts between the gene tree (Figure 2) and the species tree (Figure 1a) suffices to conclude that VEP1 has undergone multiple LGT events, and that LGT has been decisive for the evolutionary persistence of VEP1 in the face of gene loss in Bacteria.

There appear to be differences among bacteria in their propensities to be LGT donors. Of 25 LGTs, 11 involve members of the order Rhizobiales, of which seven occur in Cluster I, which is the sister cluster to land plants, and in five of these seven cases the donor is *Agrobacterium vitis*. From our data, there are not obvious differences between bacteria in their propensities to be acceptors in LGT. Besides this, Figure 3c corroborates above inferences from Figure 2 with respect to the identity of the donors in LGTs iv) and v). As to the definite bacterial identity of the donors in the remaining three interdomain LGTs, Figure 3a indicates that neither Actinobacteria, Bacteroidetes, or Betaproteobacteria could possibly be donors in LGT ii), since they received their respective VEP1 genes via lateral transfer from other members of their cluster. Accordingly, the most likely donor in LGT ii) could be a proteobacterium of either Alpha, Gamma, or Delta class, and of these most probably an alphaproteobacterium, because that class shows the oldest traceable pattern of vertical transmission. Using the same rationale, Figure 3b indicates that the most likely donor in LGT iii) could be either an actinobacterium or an alphaproteobacterium. Since an alphaproteobacterium was inferred to be the most likely donor in LGT ii), it is concluded that VEP1 should have its evolutionary origin in a proteobacterium of this class. Note that in the previous argumentation we did not consider Cluster IIb (LGT scenario in Figure 3c), because the architecture of VEP1's active site clearly indicates that this cluster is derived with respect to Clusters I and IIa (see below).

### Ecological links between VEP1-harboring taxa

LGT is expected to occur most frequently between taxa with shared habitats. If fungi, embryophytes, and trebouxiophytes each received VEP1 from bacteria living in the same or a similar environment, and the subsequent cross-bacterial LGTs occurred preferentially within the same microbial community, then the present taxonomic distribution of VEP1 in Bacteria may be biased towards members of that community. The dominant symbiosis of land plants with Fungi is the mycorrhiza, Bacteria being increasingly acknowledged as a major ecological factor for the interaction. This type of association already existed in the most recent common ancestor of Embryophyta. It currently involves the roots of ∼90% of the land plants, members of three fungal phyla, including Ascomycota, Basidiomycota, and Glomeromycota, and several bacterial taxa [80]. Figure 1b lists the bacterial groups to which the species/taxa on the left belong, colored green/red to denote presence/absence of the group in the mycorrhizosphere of mycorrhizal plants, according to [81]. Considering the bacteria that are most closely related to land plants in the VEP1 tree (i.e., Cluster I in Figure 2), there are in total 26 groups, 16 present and 10 absent in the mycorrhizosphere. Of the 16 groups that are present, nine include VEP1-containing members, whereas none of the 10 groups that are absent includes VEP1-containing members. A two-tailed Fisher's exact test yields a significant association between occurrence of VEP1 and presence of the corresponding bacterial group in the mycorrhizosphere (P = 0.0039). The association remains significant when all the bacteria of Figure 2 are included in the test (27 groups, 10 VEP1-containing of 16 mycorrhizosphere present, and 2 VEP1-containing of 11 mycorrhizosphere absent; P = 0.0473).

The dominant symbioses of green algae with fungi are the lichens. In most cases, the green algal partner is a member of the Trebouxiophyceae, which is the only branch of the Chlorophyta for which VEP1-containing species have been detected (Figure 1). About 95% of all lichen-forming fungi are ascomycetes, and the few remaining are basidiomycetes. Bacteria have recently begun to be acknowledged as third party in the lichenic symbiosis [82], [83]. The scanty data available indicate that the taxonomic composition of lichen-associated bacterial communities is dominated by groups representative of the Clusters IIa–b (Figure 2), including proteobacterial classes Alpha- [84], [85], Beta (genus *Burkholderia*; [83]), and Gamma- (genera *Pantoea*, *Pseudomonas* and *Serratia*; [86], [87]), Actinobacteria (genera *Arthrobacter*, Rhodococcus, Streptomyces; [87], [88]) and Firmicutes (genus *Paenibacillus*; [83], [87]).

### VEP1's closest remote homolog

The most distant closely related VEP1 homologs that could be identified, using pairwise sequence similarity-based BLASTp/tBLASTn tools against the NCBI's NR, EST, WGS, GSS, and HTGS databases, exhibit minimum ∼25% identity with the query. Detection of the next more-distantly related homologs, i.e. the closest remote homologs, demanded more sensitive profile and structure-based methods (see Materials and Methods). A HMM-based query of Pfam [71] and SUPERFAMILY [73] with the PRC tool [70], using the HMM profile built from this study's 81sp MSA with the HMMbuild tool at the Mobyle Portal, indicates that the VEP1 family originated from an ancient member of the NAD(P)-dependent epimerase/dehydratase family (first PRC hit, E-value 1.3e^−18^; the second hit was to the *Rmld* substrate-binding domain family, E-value 1.3e^−9^), which is one of eight different families in which Pfam classifies the currently 70 SDR protein domains in the SCOP database [72].

Table 1 lists the top 10 DaliLite [76] hits that obtain using the *D. lanata*'s *2v6f*-*g* PDB structure as query, ranked by their respective Dali Z-scores. They are the same hits that result after interrogating the Pfam [71], SUPERFAMILY [73], COG and KOG [74] databases using PSI-BLAST-based COMPASS [69], and HMM-based PRC [70], with the VEP1 sequence, this study's 81sp MSA and/or the HMM built from it as query, but for slight differences in ranking order. A PSI/SIB-BLAST search against the NCBI's NR protein sequence database yields an additional hit, namely UDP-glucuronate 4-epimerase (GAE; Table 1, last row) for which there is not a resolved structure in PDB. GAE exhibits high sequence identity (∼30%) with the fifth DaliLite hit, wbpP.

**Table 1:** Top 10 closest VEP1 remote homologs.

Official name	symbol	E.C. number	PDB code	Z-score	RMSD	lali/res	SDR family^1^	Distribution^2^
UDP-glucose 4-epimerase	UGE	5.1.3.2	2c20-A	25.7	2.8	282/329	1E	B, A, E
GDP-L-fucose synthetase	GER	1.1.1.271	1bsv-A	25.5	2.7	281/317	4E	B, A, E
GDP-4-keto-6-deoxy-D-mannose reductase	Rmd	1.1.1.281	2pk3-A	25.2	2.9	281/309	200E	B
dTDP-glucose 4,6-dehydratase	RHM	4.2.1.46	1bxk-B	24.9	3.2	282/344	2E	B, A, E
UDP-N-acetylglucosamine 4-epimerase	wbpP	5.1.3.7	1sb8-A	24.8	3.1	280/341	268E	B
CDP-tyvelose 2-epimerase	RfbE	5.1.3.10	1orr-A	24.6	3.4	287/338	148E	B
CDP-glucose 4,6-dehydratase	RfbG	4.2.1.45	1rkx-C	24.5	2.9	279/351	137E	B, A
GDP-mannose 3,5-epimerase	GME	5.1.3.18	2c59-A	24.3	2.8	282/364	93E	B, E
GDP-mannose 4,6-dehydratase	GMD	4.2.1.47	2z1m-A	24.3	3.4	293/338	3E	B, A, E
UDP-glucuronic acid decarboxylase	AXS	4.1.1.35	1z7e-D	23.4	3.4	282/644	6E	B, A, E
UDP-glucuronate 4-epimerase^3^	GAE	5.1.3.6	-	-	-	-	50E	B, A, E

1 From [48].

2 B: Bacteria; A: Archaea; E: Eukaryota.

3 PSI/SIB BLAST hit. ∼30% identical to wbpP. It lacks a resolved 3D structure in PDB.

**Figure 4:**
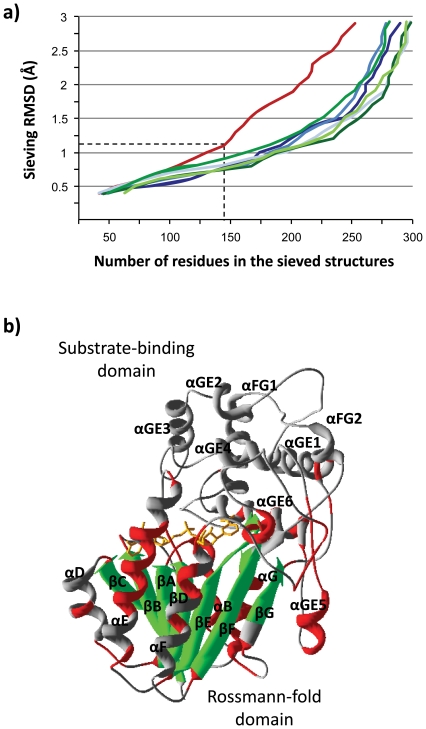
Comparative structural analysis of VEP1. a) Lesk-Hubbard plot of number of residue correspondences *vs*. RMSD for VEP1 and each of six least redundant extended SDR structures in Table 1. Each color denotes a structure with PDB code and protein name as follows: red: *2v6g*-A, VEP1; dark blue: 2c20-A, UDP-glucose 4-epimerase; medium blue: 1bsv-A, GDP-fucose synthetase; light blue: 2pk3-A, GDP-6-deoxy-D-lyxo-4-hexulose reductase; dark green: 1orr-A, CDP-tyvelose 2-epimerase; medium green: 1rkx-C, CDP-glucose 4,6-dehydratase; and light green: 2c59-A, GDP-mannose 3,5-epimerase. b) Ribbon diagram of the VEP1 (*2v6g*) structure showing the distribution of residues scoring below and above the sieving RMSD in the Lesk-Hubbard plot. The conserved core is colored red (α helices) and green (β strands). The variable regions are colored in grey. The nucleotide cofactor (NADP) is drawn in ball-and-stick representation.

The first 10 hits in Table 1 (plus GAE) belong to the Pfam's NAD(P)-dependent epimerase/dehydratase family. According to the SDR nomenclature initiative [48], the 10 hits are members of the extended type of SDRs, and each belongs into a different extended SDR family. Pairwise sequence identities between *2v6f*-*g* and each of the 10 hits are all ≤15%, which yields the hit sequences useless for the purpose of rooting the tree in Figure 2. Yet only Bacteria has representatives of all the 10 hit extended-SDR families (Table 1), which agrees with an origin of VEP1 in this domain. In order to evaluate more accurately how well *2v6f*-*g* fits into the extended type of SDRs, a standardized structural comparison was performed using the molecular sieving method at the MUSTANG-MR server [77]. This method works by identifying matching residues in a MUSTANG-generated multiple structural alignment that fit below a threshold RMSD. *2v6g* was set as the reference structure. Only Table 1 least redundant hits, i.e. exhibiting pairwise sequence identities ≤20% in the corresponding 10×10 distance matrix, were included in the analysis. Figure 4a shows the Lesk-Hubbard plot of the number of residues in the structures *vs*. their corresponding RMSDs. There is a turning point at a sieving RMSD of 1.2 Å, above which the number of superposable *2v6g* residues start to decrease rapidly compared to the extended-SDR structures of Table 1, which, however, share a nearly identical curve. Accordingly, the seven proteins in Figure 4a share a structural core of 150 residues (∼41%), outside which *2v6g* departs from the extended SDR pattern. Figure 4b shows the distribution of the VEP1 residues scoring below and above the sieving RMSD on a ribbon diagram of the *2v6g* structure. The bulk of the structurally conserved core is located towards the N-terminal side of the protein, including the central parallel β-sheet and its flanking α-helices, which constitute the Rossmann-fold scaffold for dinucleotide cofactor binding. The structurally diverging region is concentrated towards the C-terminal side of the sequence. Here, VEP1 lacks the two-stranded parallel β-sheet and the three-helix bundle that are diagnostic for the extended-SDR substrate-binding site [89], showing a fold of six α-helices instead.

### Evolution of the VEP1 active site

Early in vitro analyses showed that VEP1 exhibits the highest substrate specificity for progesterone, but could also catalyze the stereo-specific reduction of other Δ^4,5^ steroids [39], [42]. An attempt to experimentally solve the structure of a ternary enzyme-cofactor-substrate complex using progesterone was fruitless [47]. But for a initial functional assignment, using comparative sequence analysis on a limited data set [41], knowledge about the enzyme's catalytically important residues is based on *in silico* docking of the progesterone [47], [90]. Recent *in vitro* analyses have identified non-steroid substrates with which Δ^4,5^ steroid 5β-reductase achieves higher catalytic rates than with progesterone [43]. Altogether, these results challenge the generality of previous progesterone-based residue structural/catalytical assignments [47], [90]. With this caveat in mind, we'll turn to examining the patterns of variation.

**Figure 5:**
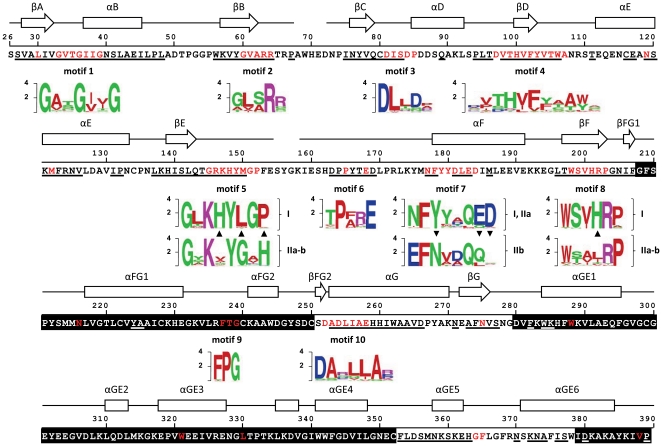
VEP1 (*2v6f*) amino acid primary sequence, secondary structural elements including α helices (arrows) and β strands (boxes), and motif logos for 10 structural/functional motifs (motifs 1–10) discussed in the text. In the primary sequence, motifs are colored red, and red residues outside motifs denote complete evolutionary conservation; the structurally conserved core in the MUSTANG-MR analysis is underlined; white/black backgrounds denote Rossmann dinucleotide-binding/substrate-binding domains, respectively. Secondary structural elements are labeled as in [47]). Motif logos were derived from the 81 sequences MSA of this study. In motif logos, green denotes a polar residue, red a hydrophobic residue, cyan a basic residues, and blue an acidic residue; arrow points denote the direction of replacements at critical sites if VEP1 arose as depicted in Figure 2. Roman numerals next to motif logos denote I: embryophytes and bacterial cluster I; IIa: fungi, trebouxiophytes, and bacterial cluster IIa; and IIb: bacterial cluster IIb. Motifs 6 and 9 are newly described in this study.

Figure 5 shows the amino acid sequence of the *2v6f* structure with secondary-structural elements included. The cofactor and substrate-binding domains are depicted on white and black sequence backgrounds, respectively. Residues constituting the structurally conserved core in the above MUSTANG-MR [77] analysis are underlined. In red are sites that are either invariant or belong to significant motifs presented as sequence logos [78] below the *2v6f* sequence. All motifs but one (motif 9) map within the Rossmann dinucleotide-binding domain, in agreement with the above MUSTANG-MR results indicating that this domain represents the bulk of the VEP1's structurally conserved core. Relative absence of recognizable motifs in the substrate-binding domain indicates divergent evolution of substrate specificity across different VEP1 homologs in Figure 2.

From motifs 1, 2, and 3 VEP1 would qualify as a prototypical extended SDR [41]. Motif 1, surrounding the N terminus of the helix αB, contains the 3 equispaced glycines fingerprint (GxxGxxG, where x denotes any amino acid), which is critical for structural integrity and binding of the diphosphate group of the dinucleotide cofactor [91]. Strict conservation of the arginine residue at the first loop position after the strand βB (Arg63) in motif 2 indicates that all the VEP1 homologs examined herein are NADPH-preferring proteins, which is relatively infrequent in extended SDRs [89], [92]. The strictly conserved Asp in motif 3, in the loop between βC and αD, is required for stabilization of the adenine-binding pocket [91], [93]. Like in extended SDRs, in VEP1 this residue is frequently followed by another charged residue two positions downchain (Asp83) [92].

In sharp contrast with motifs 1–3, which fit neatly into the known SDR cofactor-binding footprint, motif 5, in the loop from βE to αF, and motif 7, at the N terminus of αF, deviate conspicuously from the expectation for a SDR catalytic site [41], [47]. In addition, motifs 5 and 7 (and motif 8, in βF) vary in subtype-specific fashion through the tree of Figure 2. In most known SDRs, the active site contains a tetrad of catalytically important Asn, Ser (replaced by Thr in some SDRs), Tyr, and Lys residues, of which Tyr is the most conserved residue within the whole superfamily [91]. Canonical SDR active-sites were found to fit one of three alternative motifs [94]: YxxxK (classical, extended, and intermediate SDR types), YxxMxxxK (divergent), and YxxxN (complex). In VEP1 structural MSAs, the site corresponding to the SDR catalytic Tyr is at position 179 (see also [41], [47]). It is apparent that motif 7 in Figure 5 bears no similarity to any known SDR active-site. Major differences are non-conservancy of Tyr179, which is also excluded (together with Tyr180) from the structural conserved core in the MUSTANG-MR analysis, and absence of Lys at the usual position, i.e., 3 or 6 residues downchain of Tyr179. In addition, when the comparison is made against extended SDRs only, the proline typically preceding Tyr179 [94] is replaced by a Phe residue in VEP1. As to motif 5, it lacks the Ser/Thr residue of the SDR catalytic tetrad, and in position 147, which is variable in SDRs, displays a strictly conserved Lys residue. Altogether, these changes indicate that VEP1 originated through a major rearrangement of the active-site of an ancestral SDR, most likely of the extended type.

The patterns of variation at motifs 5, 7, and 8 combined indicate that, subsequently to the origin of VEP1, the novel active site underwent two additional rearrangements, coinciding with the emergence of definite groups of species in the VEP1 gene tree (Figure 2). One rearrangement occurred in the ancestral branch to the bacterial Cluster IIb, and involved the respective replacements of Asp177 and the putatively catalytic Tyr at position 179 by a Glu and an Asp residues, both of which are strictly conserved (Figure 5). On the other hand, positions 183 and 184, which are highly conserved outside Cluster IIb, evolve under comparatively relaxed constraints within this species group. The other rearrangement occurred in the ancestral branch leading to land plants and the bacterial Cluster I. The amino acids at positions 148, 151, and 201 evolved relatively free of constraints (the three positions are highly variable outside the species group of interest) until they were respectively replaced by His, Gly and His in that branch. His148 and His201 are proposed to be directly involved in the positioning of the active site for stereospecific reduction of progesterone in *D. lanata* [47]. On the other hand, residues Gly150 and His152, which are polar and are strictly conserved throughout Chlorophyta, Fungi (except for the replacement Gly150Ser in *A. nidulans*), and the bacterial Clusters IIa–b, were respectively replaced by a Leu, which is hydrophobic, and a Pro, which is an amino acid rarely involved in protein active sites [95].

Besides the aforementioned patterns supporting major active-site rearrangements in the evolution of VEP1, Figure 5 shows other patterns that either clarify previous suggested residue structural/functional roles [41], [47], [90], [91], or reveal novel putatively significant sites. Motif 4, in the central β-strand of the β-sheet (βD) and the loops connecting this strand to the previous and posterior α-helices (αD and αE), form part of the cofactor-binding pocket in *2v6g* [47], [90] and show conserved hydrophobicity. Asn119, the structural homolog of the Asn residue of the SDR catalytic tetrad (e.g., Asn111 in 17β-HSD), and also an integral element of the main dimerization interface in oligomeric SDRs (αE [91]), is strictly conserved in VEP1. Negatively charged residues in motif 10, in the αG helix, proposed to play a role in assisting the hydride transfer from the cofactor to the substrate in a duplicate of VEP1 in *Digitalis* [90], show little conservation. Two previously undescribed motifs 6 and 9 are respectively located in the loops between βE and αF, and between αFG1 and αFG2. The two motifs contain strictly conserved residues, and motif 6 is placed within the structurally conserved core by MUSTANG-MR. Since loops do not contribute much to protein core stability, motifs 5 and 6 might be important for VEP1 specific function.

## Discussion

### Widespread LGT throughout VEP1 evolution

The VEP1 gene is the outcome of a complex evolutionary history, as revealed from three main findings of this study. First, VEP1 is a member of a small-sized gene family, which exhibits a broad yet extremely patchy phyletic distribution including land plants, the green algal class *Trebouxiophyceae*, Fungi, and a few, for the most part distantly related, bacteria (Figure 1), together with a gene tree topology that depicts a polyphyly of eukaryotes nested within bacteria, and which is strongly incongruent topologically with the expected bacterial phylogeny (Figure 2). Second, VEP1 bears remote similarity to extended SDRs, but the match is limited to the NADP-binding Rossmann-fold domain (Figure 4). The gene lacks the catalytic tetrad (i.e., N-S/T-Y-K), and structurally, the substrate-binding site shows a fold of six α-helices, instead of the two stranded parallel β-sheet and the 3-α-helical bundle that are diagnostic for extended-SDRs. And third, the taxonomic composition of VEP1-harboring bacteria is biased towards taxa living in ecological association with plants –including both land plants and the trebouxiophytes— and fungi, yet plant pathogenic bacteria exclusively harbor VEP1-I.

Phylogenetic reconstruction methods can yield unexpected trees that are statistically well-supported but wrong. Frequent sources of systematic error are long branch attraction [96]–[98], and/or patterns of shared nucleotide/amino acid composition biases that contradict the phylogeny of species [99]–[101] –yet atypical codon usage or GC content patterns can be indicative of LGT [6]. It seems unlikely that phylogenetic artifacts are responsible for the overall topology of the VEP1 tree in Figure 2 because i) we used a balanced set of least redundant sequences, which should shorten most basal branches, ii) used a structure-based MSA with a high CORE index [56], iii) removed most saturated sites with Gblocks [59], iv) took among site rate variation into account in ML modeling of the process of amino acid replacement, and v) VEP1 amino acid composition departs from homogeneity by the disparity index [102], but the pattern of compositional differences across sequences can not account for the phylogenetic grouping in Figure 2 (results not shown). Besides this, VEP1 is interrupted by introns in land plants (1 intron), the trebouxiophytes (6–9), and fungi (1–4), but all intron positions are lineage-specific, which further supports that the three VEP1 eukaryotic lineages are not derived from a common eukaryotic ancestor [103].

### LGT-driven tinkering evolution of the Rossmann-fold domain VEP1 protein gene

Phenotype robustness allows for enhanced underlying genotype diversity, which in turn can facilitate exploration of the sequence space, and thus promote phenotype evolvability [30], [31], [104]. Recent studies using designability, defined as the number of sequences in a genotype space that can fold into a given structure, as a proxy to mutational robustness, found that more robust proteins evolve more functional innovations on evolutionary time [105], [106]. The NAD(P)-binding Rossmann fold is highly designable (robust), as it is capable to accommodate large structural insertions at various topological points [107], [108]. An investigation on the distribution of LGT-associated recombination breakpoints along domain-encoding sequences found that Rossmann domains do not show a tendency to be interrupted away from their centers [14]. The robustness of the Rossmann fold domain is probably related with it being organized into smaller modules, each for binding a particular region of the ligand, e.g., the glycine-rich motif for recognition of the pyrophosphate and ribose linked to the adenine ring [29] (Figure 5). Probably, these are reasons why the Rossmann-fold is one of the most ancient and widespread protein folds [109]–[115], and also one of the most promiscuous as to the number of domain partnerships (on either the N or C terminus, or interlaced [116] and functions that is capable to accommodate, being involved in a broad variety of biochemical reactions –in humans encompassing four Enzyme Commission (EC) classes, including oxidoreductases, hydrolases, lyases, and isomerases [91], [113]— and biological processes, from metabolism to regulation [94], [117], [118]. The VEP1 gene showing the Rossmann fold in combination with unprecedented active- and substrate-binding sites fits well into this scheme, suggesting that robustness, together with the significance and broad utility of providing energy/redox equivalents for catalytic reactions are features that enabled the Rossmann dinucleotide-binding domain for dissemination and evolution by the process of LGT.

Extended-SDRs have a bi-lobed three-dimensional appearance, with one lobe containing the Rossmann domain and the other lobe the substrate-binding site [92]. Yet they are considered as single-domain proteins, because at the sequence level the substrate-binding site is interspersed within the Rossman domain [119]. Likely, VEP1 is of primarily extended-SDR ancestry, since it shows a discontinuous substrate-binding site scattered among the loops of an extended-SDR-like Rossmann domain (Figure 5). This form of structural organization, together with the dramatic transformation of the ancestral secondary structure of the substrate-binding site experienced by VEP1 would be consistent with a ‘Russian Doll’ model of domain radiation [120], [121]. By this model, rapid evolution of the extended-SDR fold would primarily occur through acquisition/loss of secondary-structure based elements (e.g., α-helices, β-strands, or αβ motifs), outside the Rossmann structural core (e.g., within loops or flexible regions), rather than by stepwise accumulation of point mutations. This mode of evolution should be particularly likely in cases like VEP1, where vertical transmission is highly punctuated by long-distance LGT (see below), indicating that the gene has been frequently subjected to sequence-independent recombinational mechanisms, such as semi-homologous and illegitimate recombination (reviewed in [3]), with foreign DNAs. Along this path in VEP1, some of these changes would have eventually triggered the reassignment of important active sites, including the catalytic tetrad.

### VEP1 adds two unprecedented active sites to the SDR protein superfamily

In vitro assays using recombinant genes from plants of the genera *Arabidopsis* and *Digitalis* indicate that VEP1 has broad substrate-specificity, for it is capable of reducing a variety of substrates, including steroids and small enones, with comparable catalytic efficiencies [39], [41], [42]. Binding promiscuity appears to be common among SDRs [92]. Substrate-promiscuous SDRs are proposed to achieve this property through structural flexibility conferred on the substrate-binding site by the C-terminal loops of the proteins [122]. Since binding-promiscuous proteins can accept multiple binding partners they also have expanded actual and potential functional repertoires. Binding promiscuity should enhance the likelihood for functional recruitment of VEP1 upon LGT [123]. It may also account for why direct involvement of this protein in plant defense metabolism (see below) has been recalcitrant to proof. First comparative sequence analyses showed that VEP1 exhibits an irregular active site [41]. Subsequently, a crystallization experiment concurred with that Tyr179 residue is functionally equivalent to the catalytic Tyr residue of typical SDRs [47]. The data herein suggest that Asp119 is reminiscent of the ancestral N-S/T-Y-K catalytic tetrad, yet we found that Tyr179 is replaced by asparagine in the VEP1 protein of bacterial Cluster IIb (Figure 5), which challenges the significance of this residue in Tyr179-carrying VEP1 proteins. Tyrosine and asparagine have quite different catalytic propensities [124]. One possibility is that Tyr179 is functionally relevant, but does not play a role as critical as that played by the catalytic tyrosine in typical SDRs. Alternatively, it could be that Tyr179 plays a similar role as its putative ancestor, but the replacement Tyr179Asp forms part of a novel rearrangement of the active site in Asp179-carrying bacteria. Interestingly, the origin of Cluster IIb appears to be associated with an LGT event. VEP1 is another of an increasing number of SDRs with irregular active sites [94]. Like in some of those cases, e.g., the redox sensor proteins NmrA and HSCARG [118], [125], it may be that, in vivo, the ability of the VEP1IIb Rossmann-fold to bind dinucleotides serves a role other than catalytic.

### Propagation of VEP1 through a net of ecological interactions

If the likelihood of LGT would simply be a function of the mechanistic ease of the genetic exchange, then LGT should be more frequent between closely than distantly related taxa, because the former are more likely to be mutually compatible, i.e. sufficiently similar to undergo homologous recombination, than the later [3]. In line with the findings of other studies [21], [25], [126], we found that of the 23 interbacterial transfers shown in Figure 3a–c, 15 are long-distance transfers, implicating partners from different phyla (10 transfers) or classes (5), whilst eight implicate partners from the same proteobacterial class, the number of long-distance transfers is actually higher, if the two inter-bacterial cluster and the five bacteria-to-eukaryote transfers are taken into account (Figure 2). VEP1 is in a genome context enriched in transcriptional regulators (considering one gene on each side; Fisher's exact test P<10^−6^), which is one of two functional categories (together with defense genes) found to be enriched in long-distance LGT genes in bacteria [25]. The significance of these results, as to the relative importance of short- *vs*. long-distance LGT [21], [126] is, however, unclear, because we do not know the phylogenetic composition of the set of potential donors and recipients of the VEP1 gene in nature [127].

The negative impact of increasing genetic distance on the mechanistic ease of LGT can be offset by enhanced exposure of the partners to each others' DNAs [3], [6], [126]. Long-distance transfers should be more frequent between organisms sharing similar habitats [126]. In fact, the presence/absence distribution of the VEP1 gene agrees well with the patterns of organismal co-occurrence and life-style. The majority of the VEP1-harboring bacteria are free-living aerobic mesophiles that live in association with the dominant symbiosis of land plants with fungi (mycorrhiza), and green algae with fungi (lichen), from mutualistically, such as the free-living nitrogen fixer of the mycorrhizosphere *Gluconacetobacter diazotrophicus* PAL5, to parasitically, such as the crown-gall-causing agent *Agrobacterium vitis* S4. The order Rhizobiales appears as donor in a relatively high number of LGTs, suggesting that this lineage may serve as a hub [128], providing a medium to propagate VEP1 through plant-associated microbes. The only δ-proteobacteria in the VEP1 dataset, *Chondromyces crocatus* Cm c5 [129], is a member of the myxobacteria, which are genuinely soil-dwellers [130] typically able to lyse and feed upon other microbes, including prokaryotes and unicellular eukaryotes [131]. *C. crocatus* has been implicated in an ancient mutualistic relationship with a sphingobacterium [129], and some sphingobacteria carry the VEP1 gene (Figure 2). In two transfers implicating bacteria not known to be plant/fungi-associated, the LGT partners are, in one case, marine manganese oxidizers (from *Aurantimonas manganoxydans* SI85-9A1 to *Roseobacter sp*. GAI101; Figure 3a), and in the other case, aquatic (from *Methylibium petroleiphilum* PM1 to *Bordetella petrii* DSM 12804; Figure 3b). Most of the few remaining LGTs include transfers in which one of the partners is a generalist (e.g. *Zymomonas mobilis* ZM4, Figure 3a; *Kineococcus radiotolerans* SRS 30216; Figure 3b), hence expectedly capable of bridging between different habitats. These results are consistent with previous observations indicating that gene acquisitions are not limited to the immediate vicinity, but can be drawn from different environments [132]. Overall, our findings highlight the utility of VEP1 LGT data as a tool to investigate microbial interactions in plant/fungi-associated habitats.

Besides sharing similar environments, VEP1-harboring bacteria have in common to exhibit large genome sizes (ranging from 1,728 genes in *Zymomonas mobilis* ZM4 to 11,453 genes in *Ktedonobacter racemifer* DSM 44963). This tendency becomes more pronounced in the bacterial Cluster IIb, where all species but one (*Pantoea ananatis* LMG 20103; 4,237 genes) have genome sizes above the global median for VEP1 bearers (∼4,900 genes). This finding is in line with the observation that co-occurring genomes tend to have similar sizes [25], [127]. This association appears to be related to the fact that in Bacteria, genome size is largely determined by the amount of genes contributing to the organism lifestyle, which in turn is determined by the amount of DNA that is available for uptake by LGT from organisms living in the same habitat [25], [133]–[135]. Soil bacteria, which live in typically highly dense and diverse microbial communities, supposed to lead to strong competition for nutrients and complex interspecies communication, have also larger genomes than others [136], [137]. Cluster IIb is dominated by free-living non-obligate mycorrhizosphere-associated soil-dwellers, a condition proposed to be particularly highly demanding in terms of the required amount of genes [127], [138]. The origin of Cluster IIb is marked by the emergence of a novel form of VEP1 with a rearranged active site, hinting at the possibility of a niche-specific innovation.

### VEP1 may have been instrumental for the colonization of land by plants and fungi

The results herein suggest a plausible scenario for the formation of the VEP1 gene in an aerobic, mesophilic, and chemoorganotrophic α-proteobacterium co-inhabiting with a phylogenetically mixed microbial assemblage. Shortly after its formation, VEP1 was disseminated by LGT to surrounding microbes. The evolutionary trajectory of the gene has been highly punctuated by bursts of change apparently associated with LGT events and biological niche expansions. VEP1 crossed the domain boundary between Bacteria and eukaryotes five times. First to an ancestral fungus, probably between the time when fungi lost phagotrophy and the origin of chytrids [139]. The donor bacterium was possibly living as a free-living syntroph, or as a non-obligate host-associated symbiont with the fungus, in a fresh-water or soil habitat [139]. Then VEP1 was transferred twice independently to plant ancestors of the lineages implicated in today's two most widespread plant-fungi symbioses on Earth, mycorrhizas and lichens [140]. Considering the supposed instrumental role of mycorrhizal and lichenic associations for the colonization of the land environment by plants [139], [141], and the fact that the majority of VEP1-harboring bacteria are soil-dwellers (Figure 2), it is tempting to conclude that acquisition of VEP1 was instrumental for the terrestrialization of plants, by adding a phenotype important for life in a soil-environment (see below). In addition, it is worth noting that absence/presence of the VEP1 gene may prove an invaluable character to resolve important, yet still pending, phylogenetic issues concerning the origin of land plants, such as the exact identity of the group sister to embryophytes [142], [143]. The two most recent interdomain transfers are particularly noticeable, because there still are few examples of LGT from prokaryotes to multicellular eukaryotes [33], [37]. In both cases, the inferred donors (a common ancestor of *Geobacillus sp*. Y412MC10 and *Paenibacillus sp*. JDR-2, and an ancestral form of *Serratia odorifera* 4R×13) belong to taxa containing species known to live symbiotically with the recipient hosts (the moss *Physcomitrella patens*, and the flowering plant *Lotus japonicus*, respectively [144]–[146]). Of these two putative LGT events, the one to the moss is supported by mRNA transcript information at JGI (EntrezGene PHYDRAFT_103784). Accordingly, the *Physcomitrella* xenolog is a fragment of VEP1 with the active site motif 7-IIb (Figure 5), which forms part of a chimeric gene interrupted by two introns. Occurrence of prokaryote-derived genes in the *Physcomitrella* genome has been reported in previous studies [147], [148], the most recent one implicating a novel type of major intrinsic protein (MIP) [148].

### VEP1 hints to a LGT-based ‘Trojan Horse’ mechanism of bacterial phytopatogenesis

VEP1-harboring bacteria include non-phytopathogenic and phytopathogenic plant-associated bacteria. These two types of bacteria are not randomly distributed across the VEP1 gene tree (Figure 2): all phytopathogenic bacteria are concentrated in Cluster I, except for the two *Pantoea* strains, which are also found in Cluster IIb. This association between type of the harbored VEP1 gene and phytopathogenicity in bacteria, strongly indicates that VEP1 may be involved in the evolution of phytopathogenicity in VEP1-harboring plant pathogenic bacteria. This hypothesis can be further supported by two additional considerations. First, from the VEP1 tree (Figure 2), the most likely ancestral symbiotic state of the bacterial Cluster I is non-phytopathogenic plant-associated. Second, in land plants, VEP1 became recruited to an essential role at the interface between the host and its symbiont –perhaps, related to establishment of beneficial interactions. This second consideration is consistent with evidence from various sources: i) unlike in fungi, which exhibit a relatively high propensity for VEP1 loss, in land plants VEP1 is retained in all contemporary lineages (Figure 1), and is highly conserved [41]; ii) studies on different plant species have identified VEP1 as a defense-related gene that is induced upon wound stress [40], [44], [149], [150]; iii) a random antisense mutagenesis experiment found VEP1 to be implicated in vascular morphogenesis in *Arabidopsis* –downregulation of the gene results in reduced xylem vessels in the leaves and stems [45]; iv) a transcript-profiling assay across six developmental stages of wood formation in poplar, identified VEP1 as a candidate gene for cell wall synthesis and remodeling [151], which is in line with the fact that the closest remote homologs of VEP1 in Table 1 are all implicated in cell wall biogenesis [152]–[154]; and v) VEP1 maps within a pathogenicity island in *Xanthomonas axonopodis* pv. *citri* str. 306 (gene XAC2083 in pathogenicity island number 16 [155]), and in *C. crocatus* Cm c5 the gene is located at the downstream end of the gene cluster for the synthesis of antibiotic chondrochlorens [156]. In addition, VEP1 has been predicted to form part of the gene cluster for the synthesis of the sirodesmin phytotoxin in the plant pathogenic fungus *Leptosphaeria maculans* [157].

From the above two considerations, non-phythopathogenic plant-associated VEP1-harboring bacteria may eventually find a way to use their own encoded VEP1 gene to interfere with their host's VEP1 function to their advantage. One mechanism could be molecular mimicry. For example, the plant pathogen *Xanthomonas axonopodis pv. citri*. uses a plant natriuretic peptide-like (XacPNP) gene to modulate host homeostasis to its benefit through imitating the plant molecule [158]. In this respect, VEP1 and genes alike yet to be discovered could be considered to be potential bacterial ‘Trojan Horses’ into eukaryotes.
